# Diagnosis of pulmonary hemorrhagic leptospirosis complicated by invasive pulmonary aspergillosis complemented by metagenomic next-generation sequencing: a case report

**DOI:** 10.3389/fmed.2024.1365096

**Published:** 2024-03-04

**Authors:** Qiong-Fang Yang, Cai-Min Shu, Qiao-Ying Ji

**Affiliations:** Department of Respiratory Medicine, Affiliated Dongyang Hospital of Wenzhou Medical University, Dongyang, Zhejiang, China

**Keywords:** pulmonary hemorrhagic leptospirosis, pulmonary aspergillosis, co-infection, metagenomic next-generation sequencing, case report

## Abstract

**Background:**

Leptospirosis is a bacterial zoonosis with variable clinical manifestations. Pulmonary diffuse hemorrhagic leptospirosis often occurs rapidly and, when not promptly diagnosed and treated, it can be life-threatening. *Aspergillus flavus* is an opportunistic fungus that is commonly seen in immunosuppressed patients. Invasive pulmonary aspergillosis also progresses rapidly. This case study describes a patient with severe pneumonia caused by pulmonary hemorrhagic leptospirosis combined with invasive pulmonary aspergillosis. We have found almost no clinical reports to date on these two diseases occurring in the same patient.

**Case presentation:**

A 73-year-old male arrived at our hospital complaining of fever, general malaise, and hemoptysis that had lasted 4 days. The patient was initially diagnosed with severe pneumonia in the emergency department, but he did not respond well to empiric antibiotics. Subsequently, the patient’s condition worsened and was transferred to the ICU ward after emergency tracheal intubation and invasive ventilator. In the ICU, antibacterial drugs were adjusted to treat bacteria and fungi extensively. Although the inflammatory indices decreased, the patient still had recurrent fever, and a series of etiological tests were negative. Finally, metagenomic next-generation sequencing (mNGS) of bronchial alveolar lavage fluid detected *Leptospira interrogans* and *Aspergillus flavus*. After targeted treatment with penicillin G and voriconazole, the patient’s condition improved rapidly, and he was eventually transferred out of the ICU and recovered.

**Conclusion:**

Early recognition and diagnosis of leptospirosis is difficult, especially when a patient is co-infected with other pathogens. The use of mNGS to detect pathogens in bronchial alveolar lavage fluid is conducive to early diagnosis and treatment of the disease, and may significantly improve the prognosis in severe cases.

## Introduction

*Leptospira interrogans* is a zoonotic pathogen that is mainly transmitted by rats and domestic animals (1). Planting, slaughtering, and swimming are the risk factors for leptospirosis infection (2). Its clinical manifestations in humans are diverse and can lead to multiple organ involvement (1). *Aspergillus flavus* is a fungus that is widely distributed in the world. It is common in soil, plants, and basements, and often colonizes in the respiratory tract. It is commonly found in patients with low immune function, and can cause invasive lung disease, ear, nose and throat infection, meningitis, and other diseases (3). To the best of our knowledge, there are almost no reports of both pathogens causing the same patient to become ill at the same time. Our case report attempts to improve clinical awareness of such diseases.

## Case presentation

A 73-year-old male patient was admitted to the emergency department with the chief complaints of fever, general malaise, and hemoptysis that had lasted for 4 days. He had a history of coronary and valvular heart disease, and underwent coronary artery bypass grafting and mitral valve replacement 7 years ago in our hospital. He had recovered well after the heart surgery and had been taking aspirin enteric-coated tablets and atorvastatin calcium regularly since the surgery. Four days prior to his current visit, the patient developed fever without obvious inducement, accompanied by a small amount of hemoptysis, general fatigue, and chest tightness. The fever reached a peak of 38.3°C. The patient was diagnosed on arrival with severe pneumonia and received empirical antibiotic treatment with piperacillin/tazobactam. The patient’s condition worsened the same day to impaired consciousness and shock. Arterial blood gas analysis showed an oxygenation index of 80.98, which is significantly lower than the normal range. Therefore, emergency tracheal intubation and invasive ventilator were used to assist oxygenation. The patient was admitted to the ICU ward on the same day after stabilization.

Physical examination in the ICU showed a temperature of 36.3°C (after the use of antipyretic drugs), pulse rate 74 times/min, respiration rate 16 times/min, and blood pressure 165/115 mmHg (with norepinephrine maintained at 0.27 μg/kg. min). The patient was in a sedated state with tracheal intubation and ventilator assisted respiration, bilateral pupil diameter was 2.0 mm with no light response, bilateral lung exam showed thick breathing with audible moist rales, heart was in atrial fibrillation, abdomen was flat and soft, and both lower limbs had slight edema, with negative Babinski’s sign on both sides.

Laboratory tests showed a significant increase in neutrophils, C-reactive protein, and procalcitonin levels. Arterial blood gas analysis suggested type I respiratory failure with a PaO_2_/FiO_2_ ratio of 80.89. Blood work resulted in a white blood cell count, hemoglobin, and platelet count of 14.03 * 10^9/L, 100 g/L, and 48 * 10^9/L, respectively. Blood biochemistry indicated creatinine was 285 μmol/L, liver enzymes and direct bilirubin were slightly elevated, and pro-brain natriuretic peptide (Pro-BNP) was significantly higher than normal, suggesting heart failure (Table 1).

**Table 1:** Changes in laboratory indicators.

Test	On admission	Day 3	Day 6	Day 11	Day 16	Day21	Reference range
WBC	14.03	12.45	15.62	11.54	10.30	9.86	3.5–9.5 (10^9^/L)
CRP	229.46	114.96	14.94	5.50	1.30	4.80	<8 (mg/L)
PCT	100.00	78.40	1.51	0.34	0.13	0.08	<0.1 (ng/mL)
D-dimer	2.17	1.74	13.21	1.68	0.60	0.75	<0.5 (ug/mL)
Cr	285	370	96	79	55	56	57-111 (μmol/L)
Pro-BNP	19,604	4,683	3,235	6,044	2,948	3,442	5-125 (pg/mL)
PaO_2_/FiO_2_	81	325	217	342	370	358	400-500 mmHg
AST	48	21	14	29	34	39	9-50 (U/L)
ALT	56	17	18	25	33	36	15-40 (U/L)
CK	204	55	32	46	----	----	50-310 (U/L)
CK-MB	16	8	8	6	----	----	≤25 (U/L)
Alb	26.9	28.7	34.3	34.3	31.2	30.1	40-55 (g/L)
FIB	7.07	4.91	3.17	2.83	2.31	2.74	2-4 (g/L)
TBIL	16.5	14.4	19.2	23.0	20.2	14.2	≤26 (μmol/L)
DBIL	12.2	8.0	8.4	8.4	6.6	5.8	≤7 (μmol/L)

WBC, White blood cell count; CRP, C-reactive protein; PCT, Procalcitonin; Cr, creatinine; Pro-BNP, Pro-Brain Natriuretic Peptide; ALT, alanine aminotransferase; AST, aspartate aminotransferase; CK, Creatine kinase; CK-MB, Creatine kinase isoenzyme; Alb, Albumin; FIB, Fibrinogen; TBIL, total bilirubin; DBIL, Direct bilirubin.

**Figure 1:**
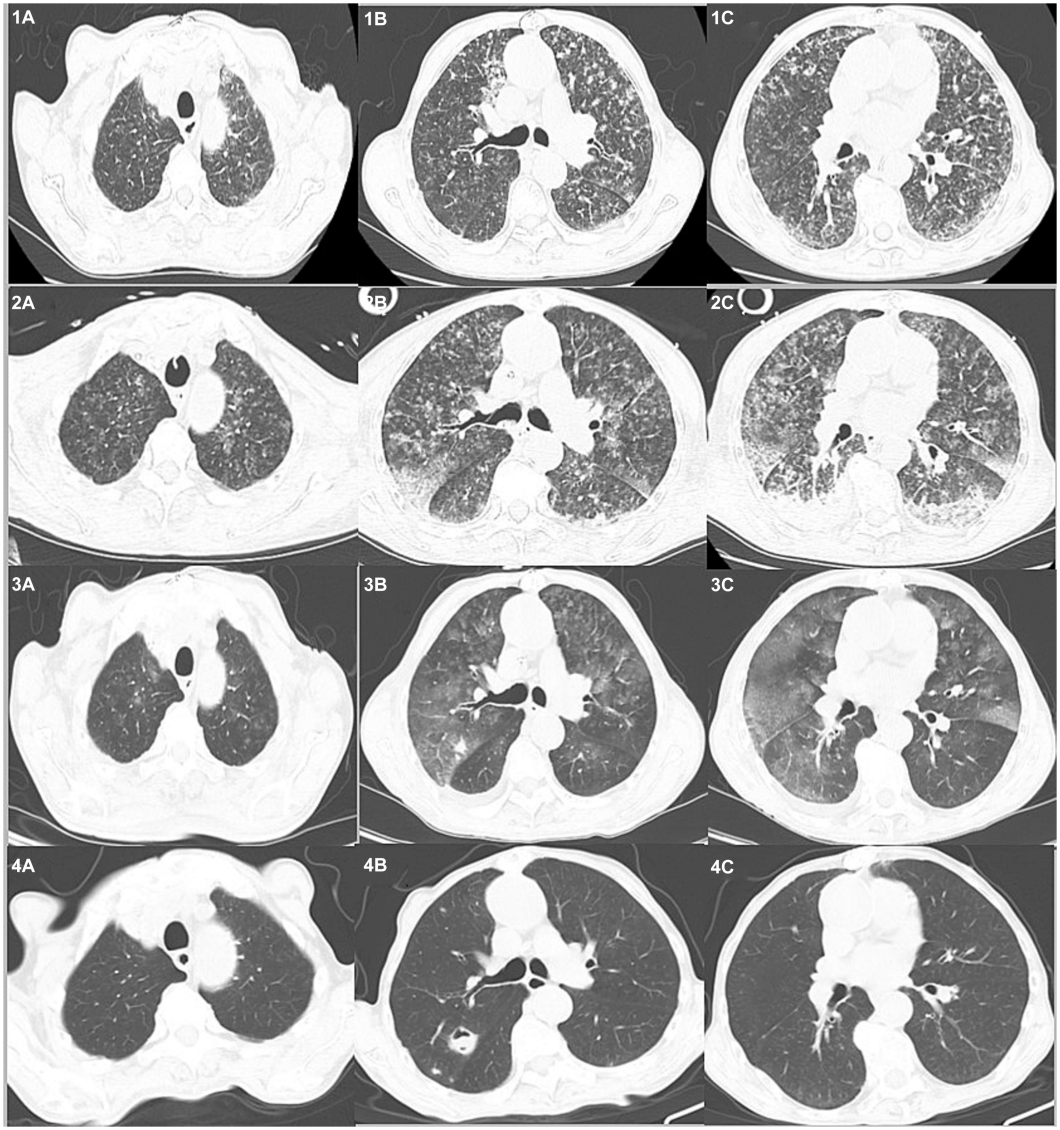
Chest CTs of the patient over time. On admission **(1A–C)** diffuse exudative lesions are evident in both lungs. Day 3 **(2A–C)** shows the progression of bilateral lung lesions. On day 17 **(3A–C)**, the bilateral exudative lesions are obviously absorbed and some nodules are enlarged. Day 40^+^ **(4A–C)** reveals the lesions are absorbed and new cavities have formed.

**Figure 2:**
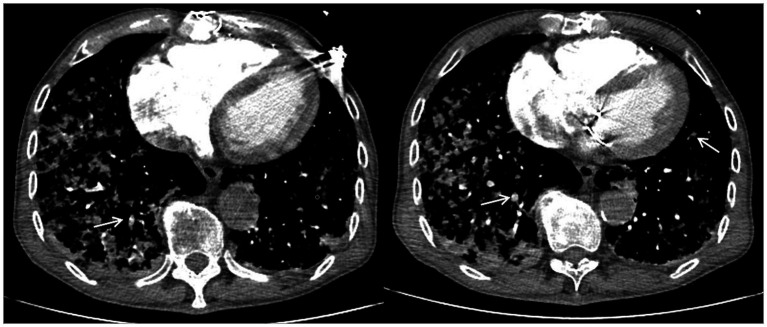
Computed tomography pulmonary angiography showing multiple pulmonary artery branch embolisms in both lungs. White arrows indicate pulmonary artery branch thrombosis.

Chest computed tomography (CT) on admission to our hospital showed diffuse exudative lesions in both lungs (Figures 1: 1A–C), and CT pulmonary angiography revealed multiple pulmonary artery branch embolisms in both lungs (Figure 2). Color Doppler ultrasound of the heart showed decreased left ventricular systolic function, enlarged right heart, and a cardiac ejection fraction of 45%. Lower limb vascular Doppler ultrasound did not indicate deep vein thrombosis. Bedside electrocardiogram revealed atrial fibrillation with a rapid ventricular rate of 114 times/min.

To detect the etiology of the pneumonia, we performed a series of microbiologic diagnostics including staining and culture and serologic testing for viruses, bacteria, and fungi, all of which were negative (Table 2).

**Table 2:** Pathogenic test results.

Test	Results	Reference range
Blood culture	Negative	Negative
Sputum culture	Negative	Negative
Cryptococcus antigen	Negative	Negative
Fungus-D glucan determination	<37.50 pg./mL	≤70
Galactomannan determination	0.26	≤0.5
Sputum smear for acid-fast bacilli	Negative	Negative
Sputum tuberculosis culture	Negative	Negative
Anti-HIV	Negative	Negative
COVID-19 antigen	Negative	Negative

After the patient was transferred to the ICU, due to the consideration of severe community-acquired pneumonia, the condition progressed rapidly, and the initial anti infection treatment failed. Moreover, the pathogen of the infection was not clear, so he was given imipenem and cilastatin sodium (1.0 g, I.V. infusion, q12h), vancomycin (0.5 g, I.V. infusion, qd), and voriconazole (0.2 g, I.V. infusion, qd). The dosage of antibiotics was based on the creatinine clearance rate. The patient was also given methylprednisone (40 mg, I.V., q12h) as an anti-inflammatory, lyophilized recombinant human brain natriuretic peptide (0.0075 μg/kg. min, I.V. infusion) to treat heart failure, and nadroparin calcium (4,100 IU, I.H., q12h) for anticoagulation after platelet count recovery.

**Figure 3:**
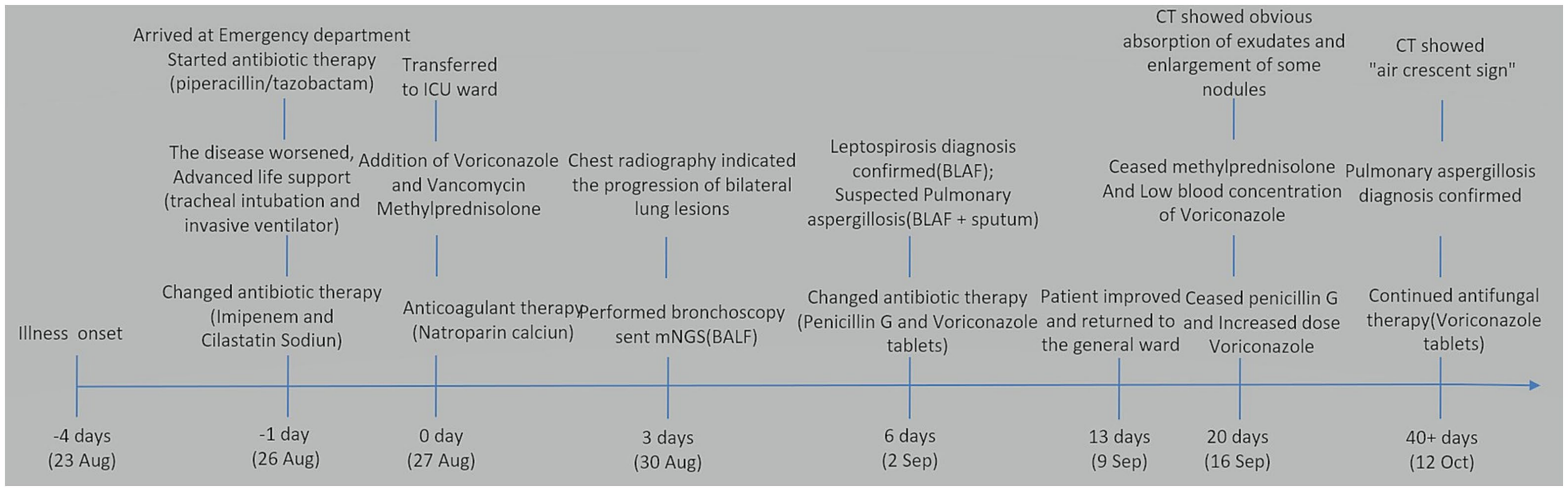
Hospitalization timeline and clinical treatments.

After 3 days, the blood inflammation indices decreased, but the patient still had current fever. Chest CT reexamination showed the progression of bilateral lung lesions, as illustrated in Figures 1: 2A–C, and so bedside bronchoscopy was given. Bronchoscopy displayed diffuse blood infiltration and mucosal swelling in the left and right bronchial lumens. The bronchial alveolar lavage fluid was sent for mNGS examination. Three days later, mNGS showed seven sequence numbers of *Leptospira interrogans* and 1,289,906 sequences of *Aspergillus flavus*. A culture of the bronchial alveolar lavage fluid revealed *Aspergillus flavus*: 1+. Therefore, in consideration of pulmonary hemorrhagic leptospirosis complicated with pulmonary aspergillosis, administration of vancomycin, imipenem cilastatin, and voriconazole injection was stopped, and the patient was given penicillin G (400,000 U I.V. infusion, q4h) combined with voriconazole tablets (0.2 g, p.o., q12h). After using penicillin G for a week, the patient’s condition improved and he was returned to the general ward. The chest CT reexamination on day 17 showed that the exudative lesions in bilateral lungs were obviously absorbed, and some nodules were enlarged, as shown in Figures 1: 3A–C. Inflammation indices in the blood were normal, so penicillin sodium was stopped. The enlarged pulmonary nodules were considered to be associated with *Aspergillus flavus* infection, so the blood concentration of voriconazole was detected. The detection result was 1.0 mg/L (the reference value range was 0.5–5 mg/L), which was at the normal lower limit level. Therefore, voriconazole was increased to 250 mg once every 12 h for antifungal treatment. Methylprednisolone was gradually reduced to stop, and the patient was discharged with voriconazole tablets. Figure 3 illustrates the timeline of hospitalization and clinical treatments, and Table 1 shows the changes in relevant laboratory indicators during the course of the disease. Chest CT reexamination 3 weeks after discharge showed that the bilateral lung lesions were obviously absorbed, and there were new cavities, as shown in Figures 1: 4A–C. The images showed an air crescent sign typical of aspergillosis. Therefore, the patient continued to receive oral antifungal treatment with voriconazole tablets for 3 to 6 months.

## Discussion and conclusions

Leptospirosis is a zoonosis caused by the *Leptospira interrogans* bacteria. Its incidence is on the rise globally, and is 10 times higher in tropical areas than in temperate regions (4). Transmission of the organism to humans occurs via portals of entry such as cuts or abraded skin, mucous membranes, or conjunctivae (5). Human exposures that lead to infection include contact with urine-contaminated soil or water (e.g., floodwater, ponds, rivers, streams, and sewage), ingestion of food or water contaminated by urine, or direct contact with the urine or reproductive fluids from infected animals (6). The patient of our case study was a farmer who had a history of drinking stream water and bathing in the wild. It was considered that he might have been infected with *Leptospira* by contacting and drinking contaminated water before he became ill. The onset of the patient’s illness was at the end of August, which is consistent with the seasonal occurrence of leptospirosis in Asia. Most cases occur from July to October, with the peak in August and September each year (7). In addition, this farmer had been exposed to mold in foods such as cereals and beans. We theorized that *Aspergillus flavus* had colonized in his respiratory tract for a long time, and that the continuous use of hormones during the treatment of leptospirosis led to the final growth of the fungus.

Leptospirosis can be categorized as non-icteric or icteric, and its clinical symptoms are variable. Most non-icteric cases are mild and self-limited or asymptomatic, while the icteric type is severe and potentially fatal (8). In the acute stage of leptospirosis, there are many nonspecific symptoms such as fever, muscle soreness, headache. The icteric phase is distinguished by significantly raised serum bilirubin levels. Also, very few patients have rapidly progressive pulmonary hemorrhage and respiratory distress, but this manifestation is often seen in icteric leptospirosis (9).

Invasive pulmonary aspergillosis is mainly characterized by fever, cough, hemoptysis, and progressive dyspnea (10) and is impossible to distinguish from leptospirosis based only on clinical manifestations. Although the patient in our case study had non-icteric leptospirosis, because of the co-infection with *Aspergillus flavus*, he had severe pulmonary hemorrhage, and rapidly progressed to acute respiratory distress syndrome, ultimately needing emergency tracheal intubation. Therefore, when it is difficult to identify the pathogen based on clinical symptoms, more attention should be paid to detailed medical history collection.

Imaging of pulmonary diffuse hemorrhagic leptospirosis typically reveals bilateral patchy peripheral infiltrates, often with a “snowflake” appearance, that can progress to confluent consolidation or a ground-glass appearance (11). The lesions are obvious in the middle and lower lobes of bilateral lungs, and the imaging manifestations are consistent with the clinical symptoms. After timely treatment, the lung lesions mostly disappear within a week.

Early imaging of invasive pulmonary aspergillosis is characterized by a halo sign, which represents hemorrhagic nodules (10). Our patient was initially diagnosed with leptospirosis and the possibility of aspergillus infection was considered. After standard treatment with penicillin G, the exudative lesions in the lung were obviously absorbed, but some nodules were still enlarged. The enlarged nodules further supported a diagnosis of aspergillus infection. Finally, after 4 weeks, the lung imaging showed an air crescent sign typical of aspergillus infection. It is difficult to distinguish overlapping infections of leptospirosis and aspergillosis from imaging alone. Misdiagnosis of overlapping syndromes can lead to delayed diagnosis and treatment, which further emphasizes the importance of detailed inquiry of the patient’s epidemiological history.

*Leptospira* is typically identified by molecular, serological, or culturing methods. Polymerase chain reaction (PCR), modified agglutination test, enzyme linked immunosorbent assay, and mNGS can all be used for early diagnosis of leptospirosis (12), but traditional detection methods have their shortcomings. For example, although PCR provides rapid results, its overall sensitivity is relatively low (13). Moreover, traditional methods of detecting leptospirosis can only help confirm an existing diagnosis. Use of mNGS can not only help confirm the diagnosis, but also provide important diagnostic clues for patients with atypical symptoms or with multiple overlapping infections (14). At the initial disease stage in our case study, the empirical effect of anti-infection measures was not good, and the routine pathogenic test results were negative. Finally, mNGS confirmed the presence of both *Leptospira* and *Aspergillus flavus*. From this it can be concluded that if the clinical symptoms are serious and the pathogen is not clear, the early use of mNGS to detect pathogens can buy valuable time for saving lives.

Current recommendations in domestic and foreign literature suggest that patients with leptospirosis lung involvement have good clinical reactions to penicillin, third-generation cephalosporins, meropenem, and fluoroquinolone antibiotics. For patients with a clear diagnosis of severe leptospirosis, penicillin and hormones are recommended for symptomatic treatment (15). However, when our patient was given anti-infective treatment with piperacillin/tazobactam on admission, his clinical symptoms worsened, resulting in the need for tracheal intubation. After comprehensive analysis, we concluded that the patient had a Herxheimer reaction. This reaction is an aggravation of clinical symptoms such as high fever, chills, myalgia, increased heart rate and respiration, and decreased blood pressure, and it is caused by the release of toxins during the treatment with penicillin or other antibacterial drugs (16). Following a Herxheimer reaction, symptomatic supportive treatment is the main approach (16). Penicillin can be gradually increased in small doses, supplemented by glucocorticoids, and the symptoms are usually relieved quickly. For our patient, we changed to penicillin G supplemented by glucocorticoid and respiratory support, and the condition gradually stabilized. Voriconazole is the first choice of medication for pulmonary aspergillus infection (3). The patient was given voriconazole for antifungal treatment at the beginning of the disease. The conventional dose did not reach the expected clinical efficacy, but the final focus absorption was good after an appropriate dosage increase.

In conclusion, the causes of respiratory tract infections are complex and diverse, with nonspecific clinical symptoms. Therefore, clinicians cannot ignore the importance of epidemiological history during medical intake. In addition, the new diagnostic technology mNGS is helpful for the early diagnosis of patients with severe overlapping infection. When the clinical efficacy of treatment for a single pathogen is inconsistent with the changes in lung imaging, the possibility of co-infection should be considered to reduce missed diagnosis and misdiagnosis.

## Data availability statement

The original contributions presented in the study are included in the article/supplementary material, further inquiries can be directed to the corresponding author.

## Ethics statement

Written informed consent was obtained from the individual(s) for the publication of any potentially identifiable images or data included in this article.

## Author contributions

Q-FY: Data curation, Formal analysis, Investigation, Methodology, Writing – original draft, Writing – review & editing. C-MS: Supervision, Writing – review & editing. Q-YJ: Writing – review & editing.
